# Sex-based disparities in cardiovascular outcomes: real-world evidence following chimeric antigen receptor T-cell therapy

**DOI:** 10.3389/fcvm.2026.1838296

**Published:** 2026-07-08

**Authors:** Abdul Rasheed Bahar, Yasemin Bahar, Paawanjot Kaur, Nagasai Yalavarthi, Ali Awad, Shaheena Raheem, Ahmet Afsin Oktay

**Affiliations:** 1 Department of Medicine, Wayne State University/ Detroit Medical Center, Detroit, MI, United States; 2 The Heart and Vascular Institute, Rush University Medical Center, Chicago, IL, United States

**Keywords:** cardio-oncology, cardiovascular outcomes, chimeric antigen receptor T-cell therapy, sex-based disparities, TriNetX

## Abstract

**Background:**

Cardiovascular complications are increasingly recognized following chimeric antigen receptor T-cell (CAR-T) therapy, yet potential sex-based differences in cardiovascular risk remain incompletely defined. We evaluated sex-based differences in major adverse cardiovascular events (MACE) and cardiovascular complications following CAR-T therapy.

**Methods:**

Using the TriNetX Global Research Network, we identified adults treated with CAR-T therapy between 2015 and 2025. Male and female patients were propensity score–matched 1:1 on baseline characteristics. The primary outcome was MACE, defined as myocardial infarction, stroke, or all-cause mortality, assessed at 1- and 2-year follow-up. Secondary outcomes included all-cause mortality and other cardiovascular complications. Outcomes were compared using risk ratios and Cox proportional hazards models.

**Results:**

Among 4,944 matched patients (2,472 males and 2,472 females), baseline characteristics were well balanced. At 1 year, males had a higher incidence of MACE compared with females (558 vs. 437 events; RR: 1.22, 95% CI: 1.09–1.36), with a higher hazard on time-to-event analysis (HR: 1.22, 95% CI: 1.08–1.38). This association persisted at 2 years (697 vs. 593 events; RR: 1.15, 95% CI: 1.05–1.26; HR: 1.18, 95% CI: 1.06–1.32). At 2 years, males also had a higher risk of all-cause mortality and higher risks of atrial fibrillation, ventricular arrhythmias, high-grade atrioventricular block, and pericarditis.

**Conclusions:**

Male sex was associated with a higher risk of MACE following CAR-T therapy, along with a greater burden of arrhythmic and conduction abnormalities. These findings highlight the importance of incorporating biological sex into cardiovascular risk assessment and monitoring strategies in patients undergoing CAR-T therapy.

## Introduction

Chimeric antigen receptor T-cell (CAR-T) therapy has resulted in durable remissions in relapsed or refractory B-cell malignancies by redirecting autologous T cells against tumor-associated antigens, most notably CD19. These landmark clinical successes have established CAR-T therapy as a transformative immunotherapeutic approach that is now routinely used worldwide for relapsed or refractory B-cell malignancies and multiple myeloma, with multiple products approved by the U.S. Food and Drug Administration and European Medicines Agency (1, 2).

CAR-T cell therapy has expanded into routine clinical practice, achieving complete remission rates of 52 to 90% and durable responses up to 50% at one year with refractory B-cell malignancies. As a result, CAR-T therapy has shifted many patients from a predominantly palliative trajectory to one characterized by long-term survival. Consequently, increasing attention has been directed toward treatment-related toxicities beyond cytokine release syndrome (CRS) and neurotoxicity, particularly acute and chronic cardiovascular complications (3). Patients receiving CAR-T cell therapy often have significant baseline cardiopulmonary comorbidity due to prior therapies, yet remain eligible for treatment due to functional status (4). Emerging evidence from real-world cohorts and expert consensus statements has identified heart failure, arrhythmias, conduction abnormalities, and major adverse cardiac events as clinically relevant sequelae of CAR-T therapy, underscoring the need for systematic cardiovascular risk assessment and surveillance in this population (5–7). Although cardiovascular complications of CAR-T therapy are increasingly recognized, potential sex-based differences in susceptibility, presentation, and outcomes remain poorly defined. Established biological differences in immune activation, inflammatory signaling, and cardiovascular remodeling between males and females may contribute to differential cardiotoxic risk profiles following immune-based cancer therapies, highlighting an important gap in current cardio-oncology evidence (8, 9).

Unlike conventional cardiotoxic cancer therapies, CAR-T cell therapy induces a distinct immunologic state characterized by rapid T-cell expansion, profound cytokine release, endothelial activation, and systemic inflammatory stress, with CRS directly implicated in arrhythmias, myocardial dysfunction, and conduction abnormalities. These mechanisms differentiate CAR-T–associated cardiotoxicity from that observed with chemotherapy or immune checkpoint inhibitors and may interact with well-described sex-based differences in immune and inflammatory signaling and cardiovascular remodeling (3). In this context, we conducted a large real-world, propensity score–matched analysis to evaluate sex-based differences in cardiovascular outcomes following CAR-T cell therapy. By examining short- and intermediate-term cardiotoxic events in a contemporary multicenter cohort, this study addresses an important gap in the cardio-oncology literature and provides novel insights to inform sex-specific cardiovascular risk stratification and surveillance strategies.

## Methods

### Study design and data source

This retrospective cohort study was conducted using the TriNetX Global Research Network, a federated electronic health record database comprising data from academic and community healthcare organizations. The database includes deidentified patient-level information on demographics, diagnoses, procedures, medications, and clinical outcomes derived from routine care. The database integrates demographic information, diagnoses, procedures, medications, laboratory measurements, and outcomes, standardized using the Observational Medical Outcomes Partnership (OMOP) common data model and coded with established terminologies including ICD-10, CPT, and RxNorm (10). All data are deidentified in accordance with the Health Insurance Portability and Accountability Act (HIPAA) Privacy Rule. Because this study involved analysis of deidentified, aggregated data, it was determined to be exempt from institutional review board oversight by the Wayne State University Institutional Review Board. The TriNetX platform complies with the Health Insurance Portability and Accountability Act (HIPAA) and the General Data Protection Regulation (GDPR) (10). The study was conducted in accordance with the principles of the Declaration of Helsinki and is reported in compliance with the Strengthening the Reporting of Observational Studies in Epidemiology (STROBE) guidelines (11).

### Study population

We identified adult patients aged ≥18 years who received chimeric antigen receptor T-cell (CAR-T) therapy for underlying hematologic malignancies using International Classification of Diseases, Tenth Revision (ICD-10) diagnosis and procedure codes within the TriNetX Global Research Network (Central Illustration). For the present analysis, the TriNetX database was queried from January 1, 2015, through November 30, 2025. The receipt of CAR-T therapy served as the index event, and patients were included only following real-world adoption of CAR-T therapy after FDA approval. Patients were subsequently followed from the index event, with outcomes assessed at 1- and 2-year timepoints. Patients were categorized into two cohorts based on documented biological sex (male or female) as recorded in the electronic health record. To enhance exposure specificity and minimize misclassification, CAR-T therapy was defined using a new-user design, with the index date corresponding to the first documented administration of CAR-T therapy. All cardiovascular outcomes were defined as incident events occurring after the index date.

**Central Illustration:**
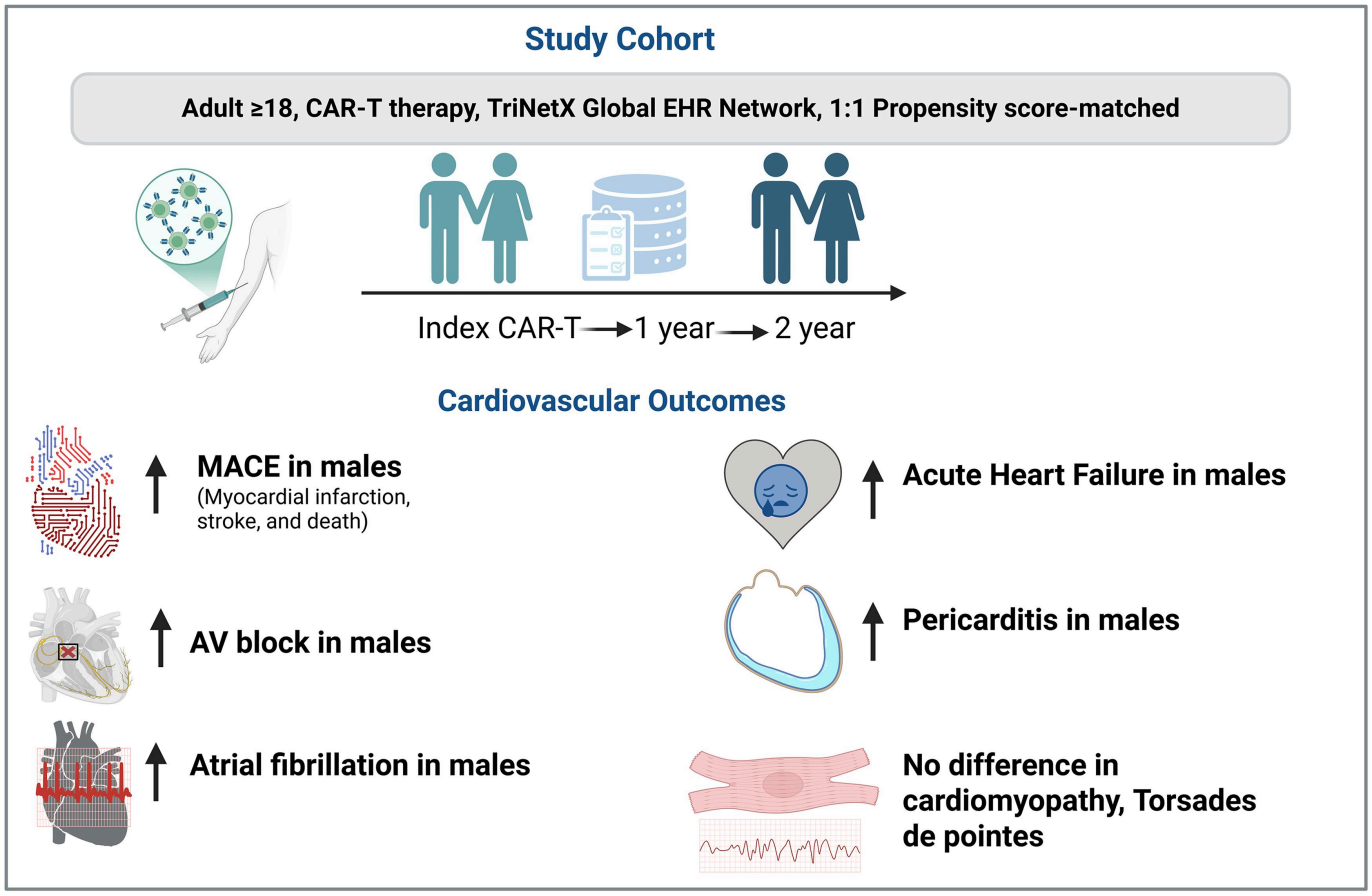
Cardiovascular complications after CAR-T cell therapy: sex-based disparities.

### Study endpoints

The index date was defined as the date of CAR-T cell therapy administration. Cardiovascular outcomes were assessed at 1-year and 2-year follow-up after the index date. The primary outcome was major adverse cardiovascular events (MACE), defined as a composite of myocardial infarction (MI), stroke, and all-cause mortality. Secondary outcomes included all-cause mortality, MI, stroke, acute heart failure, atrial fibrillation, ventricular arrhythmias, torsade de pointes, high-grade atrioventricular (AV) block (referring to Mobitz II or complete AV block), cardiomyopathy, and pericarditis. All outcomes were identified using standardized ICD-10 diagnostic codes recorded after the index date.

### Statistical analysis

Baseline characteristics were summarized using descriptive statistics, with continuous variables reported as mean ± SD and categorical variables as counts with percentages. Covariate balance before and after matching was assessed using standardized mean differences (SMDs), with an SMD <0.10 indicating adequate balance. To account for baseline differences between male and female patients, 1:1 propensity score matching was performed using a nearest-neighbor algorithm with a caliper of 0.1 of the pooled SD of the logit of the propensity score. Propensity scores were estimated using multivariable logistic regression incorporating demographic variables, available cancer-related characteristics, baseline cardiovascular comorbidities, baseline medication use, prior radiotherapy exposure, and CRS severity and ICANS recorded before or at the index date. Time-to-event analyses were performed for MACE using Kaplan–Meier survival curves and Cox proportional hazards regression, with results reported as hazard ratios (HRs) and 95% confidence intervals (CIs). The proportional hazards assumption was assessed using the Schoenfeld residuals test as implemented within the TriNetX platform. In addition, outcomes at 1-year and 2-year follow-up were compared between matched cohorts using risk ratios (RRs) with corresponding absolute risk differences and 95% CIs. Two-sided *P* values <0.05 were considered statistically significant for the primary outcome, whereas secondary outcomes were interpreted as exploratory and no correction for multiple comparisons was performed. As a sensitivity analysis, MACE was redefined as a composite of MI and stroke only, excluding all-cause mortality, to assess whether the primary findings were driven by non-cardiovascular deaths. Additionally, E-values were calculated for the primary and selected secondary outcomes to quantify the minimum strength of association that an unmeasured confounder would need to have with both sex and the outcome to fully explain the observed associations. Larger E-values indicate that substantial unmeasured confounding would be required to negate the observed effects (12). All analyses were conducted using the integrated TriNetX analytics platform with R-based statistical methods, and all tests were two-sided.

## Results

### Study population

**Figure 1:**
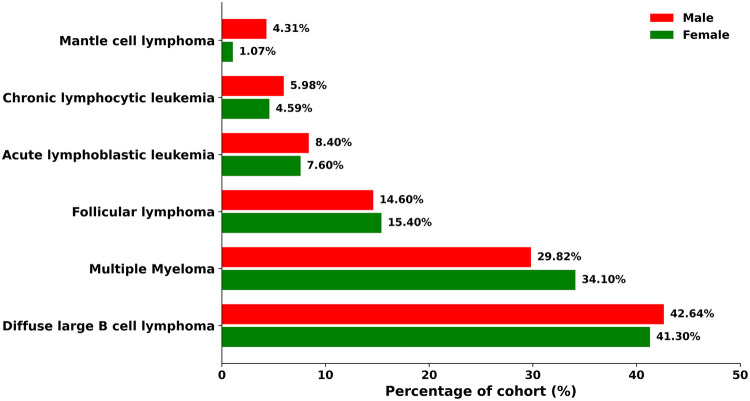
Baseline cancer types in patients undergoing CAR-T therapy.

A total of 6,488 adult patients who received CAR-T cell therapy were identified prior to propensity score matching, including 3,979 males and 2,509 females. After 1:1 propensity score matching, the final analytic cohort consisted of 4,944 patients, with 2,472 males matched to 2,472 females. Before matching, several baseline demographic and clinical characteristics differed between male and female patients. Figure 1 illustrates the distribution of baseline cancer types across the two cohorts. After propensity score matching, baseline characteristics were well balanced between the two cohorts. Most covariates achieved SMD <0.10, indicating adequate covariate balance; however, a small number of variables had residual SMDs between 0.10 and 0.20 (Table 1).

**Table 1A:** Baseline characteristics of the study cohorts before and after propensity score matching.

Baseline characteristics	Before propensity matching			After propensity matching		
	Male *N* = 3,979	Female *N* = 2,509	*P*-value	Male *N* = 2,472	Female *N* = 2,472	SMD
Demographics						
Age (Mean ± SD)	60.4 +/− 15.2	59.9 +/− 15.5	0.188	59.9 +/− 15.2	59.9 +/− 15.6	<0.001
White	3,068 (77.1%)	1,823 (72.6%)	<0.001	1,847 (74.7%)	1,815 (73.4%)	0.029
African American	348 (8.75%)	294 (11.7%)	<0.001	255 (10.3%)	271 (10.9%)	0.020
Asian	106 (2.6%)	77 (3.1%)	0.337	63 (2.5%)	76 (3.1%)	0.031
American Indian	15 (0.3%)	11 (0.4%)	0.702	12 (0.4%)	11 (0.4%)	0.005
Native Hawaiian/ Pacific Islander	10 (0.2%)	10 (0.4%)	0.297	10 (0.4%)	10 (0.4%)	<0.001
Diagnoses						
Essential hypertension	2,022 (50.8%)	1,156 (46.1%)	<0.001	1,086 (43.9%)	1,136 (45.9%)	0.040
Hyperlipidemia	1,353 (34%)	754 (30.1%)	<0.001	701 (28.3%)	742 (30.1%)	0.036
Obesity	786 (19.7%)	578 (23%)	<0.001	513 (20.7%)	547 (22.1%)	0.033
Type 2 diabetes mellitus	788 (19.8%)	444 (17.7%)	0.035	428 (17.3%)	437 (17.6%)	0.009
Liver disease	585 (14.7%)	382 (15.2%)	0.564	340 (13.7%)	377 (15.2%)	0.042
CKD	666 (16.7%)	318 (12.6%)	<0.001	308 (12.4%)	317 (12.8%)	0.010
Atrial fibrillation/flutter	526 (13.2%)	212 (8.45%)	<0.001	296 (11.9%)	204 (8.25%)	0.123
Obstructive sleep apnea	489 (12.2%)	197 (7.8%)	<0.001	286 (11.5%)	191 (7.7%)	0.130
Heart Failure	469 (11.7%)	247 (9.8%)	0.015	255 (10.3%)	242 (9.7%)	0.017
CAD	729 (18.3%)	239 (9.5%)	<0.001	223 (9.0%)	239 (9.6%)	0.022
History of MI	170 (4.2%)	65 (2.5%)	<0.001	65 (2.6%)	65 (2.6%)	<0.001
Cerebral Infarction	121 (3.0%)	80 (3.2%)	0.738	58 (2.3%)	80 (3.2%)	0.054
Peripheral vascular diseases	113 (2.8%)	70 (2.7%)	0.905	55 (2.2%)	69 (2.7%)	0.036
Active Tobacco use	118 (2.9%)	50 (1.9%)	0.016	49 (1.9%)	50 (2.0%)	0.002
Medications						
Beta blockers	1,473 (37%)	929 (37%)	0.995	870 (35.1%)	900 (36.4%)	0.025
Statins	1,360 (34.1%)	694 (27.6%)	<0.001	659 (26.6%)	689 (27.8%)	0.027
Anthracyclines	960 (24.1%)	599 (23.8%)	0.816	569 (23.0%)	592 (23.9%)	0.021
ACEi	867 (21.7%)	474 (18.8%)	0.005	460 (18.6%)	470 (19.0%)	0.010
ARBs	646 (16.2%)	416 (16.5%)	0.714	379 (15.3%)	397 (16.0%)	0.020
Trastuzumab	10 (0.2%)	10 (0.4%)	0.297	10 (0.4%)	10 (0.4%)	<0.001
VEGF/VEGFR Inhibitors	15 (0.3%)	14 (0.5%)	0.28	10 (0.4%)	14 (0.5%)	0.023
Radiation therapy	533 (13.8%)	292 (11.8%)	0.36	309 (12.7%)	303 (12.4%)	0.01
Laboratory (Mean ± SD)						
eGFR	85 ± 35	83 ± 36	0.04	86 ± 35	83 ± 36	0.08
Troponin	5.9 ± 5	4.6 ± 3	0.15	4.8 ± 3	4.7 ± 3	0.10
BNP	185 ± 549	146 ± 360	0.22	184 ± 571	149 ± 365	0.07
LVEF (%)	58.4 ± 7.3	60 ± 7.8	0.001	58.7 ± 7.1	60 ± 7.9	0.17
HbA1c	6 ± 1.2	5.8 ± 1	0.009	6 ± 1.3	5.8 ± 1	0.14
LDL	90 ± 41	101 ± 37	<0.001	93 ± 44	101 ± 38	0.17
Triglyceride	167 ± 214	162 ± 290	0.64	166 ± 249	163 ± 296	0.01

CKD, chronic kidney disease; CAD, coronary artery disease; ACEi, angiotensin converting enzyme inhibitors; ARBs, angiotensin receptor blockers; VEGF, vascular endothelial growth factor; EGFR, endothelial growth factor receptor.

**Table 1B:** Baseline CRS and ICANS.

Variable	Before propensity matching			After propensity matching		
	Male (*N* = 3,979)	Female (*N* = 2,509)	*P*-value	Male (*N* = 2,472)	Female (*N* = 2,472)	SMD
CRS Grade I	323 (8.4%)	191 (7.7%)	0.79	203 (8.3%)	194 (8.0%)	0.004
CRS Grade II	131 (3.4%)	85 (3.4%)	0.08	105 (4.3%)	93 (3.8%)	0.017
CRS Grade III	33 (0.9%)	16 (0.6%)	0.26	15 (0.6%)	14 (0.6%)	0.010
CRS Grade IV	10 (0.3%)	10 (0.4%)	0.31	10 (0.4%)	10 (0.4%)	0.001
CRS Grade V	10 (0.3%)	10 (0.4%)	0.32	10 (0.4%)	10 (0.4%)	0.012
CRS Grade Unspecified	122 (3.2%)	69 (2.8%)	0.62	84 (3.4%)	78 (3.2%)	0.021
ICANS	71 (1.8%)	55 (2.3%)	0.29	48 (1.9%)	50 (2.1%)	0.01

CRS, cytokine release syndrome; ICANS, immune effector cell-associated neurotoxicity syndrome.

### Primary outcome

**Figure 2:**
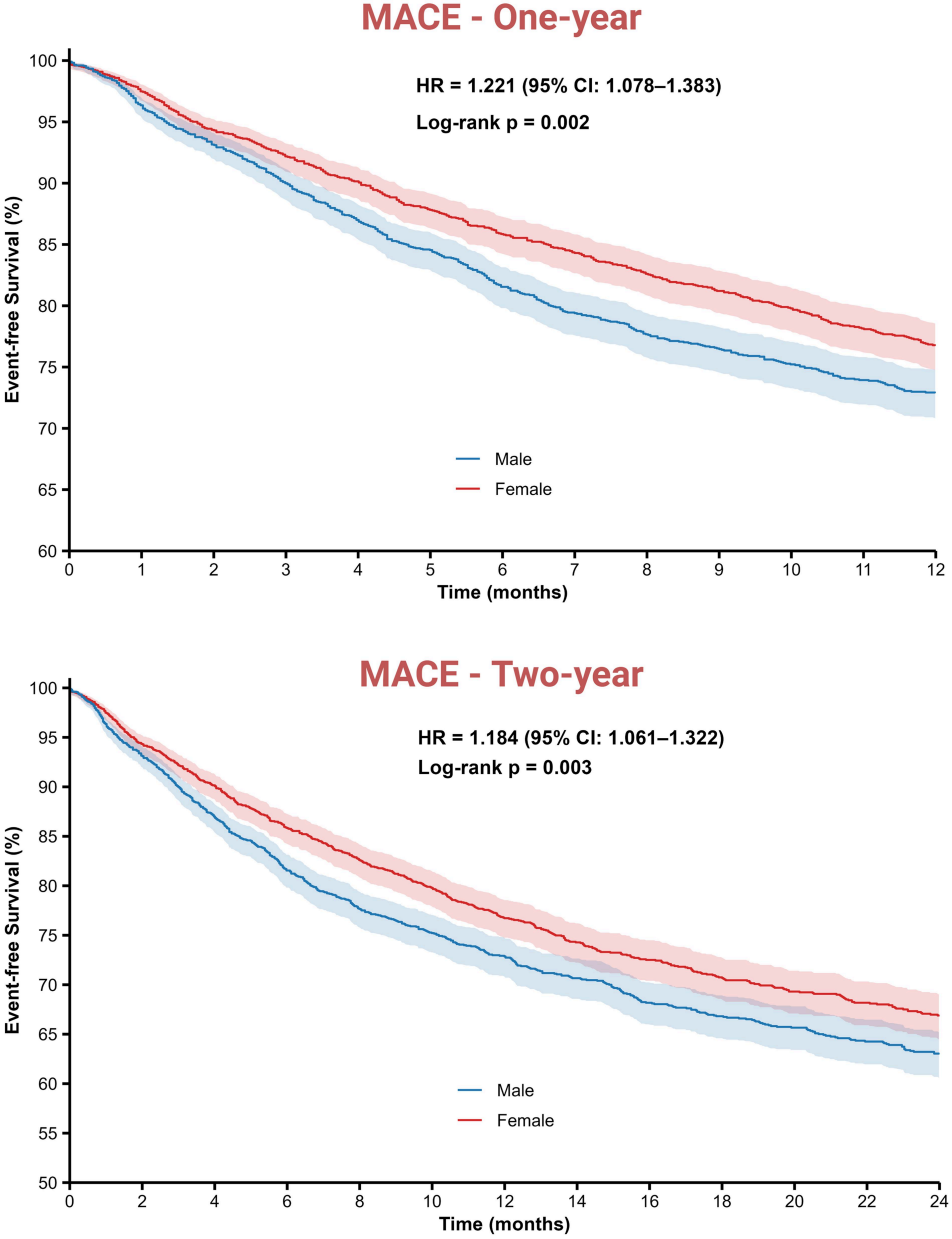
Sex differences in MACE following CAR-T therapy at 1 and 2 years. Kaplan–Meier curves for event-free survival from MACE in propensity-matched male and female patients treated with CAR-T therapy. Panel shows 1-year outcomes and Panel shows 2-year outcomes. Male patients demonstrated a higher hazard of MACE compared with female patients at both time points. Shaded areas represent 95% confidence intervals.

At 1 year, the incidence of MACE was significantly higher among male patients compared with female patients [558 vs. 437 events; absolute risk difference +4.96%; risk ratio (RR) 1.22, 95% CI: 1.09–1.36; *P* = 0.003]. Consistent with these findings, time-to-event analysis demonstrated a higher hazard of MACE among males compared with females [hazard ratio (HR) 1.22, 95% CI: 1.08–1.38; log-rank *P* = 0.002] (Figure 2). This association persisted at 2-year follow-up, with males continuing to experience a higher incidence of MACE compared with females (697 vs. 593 events; absolute risk difference +4.21%; RR 1.15, 95% CI: 1.05–1.26; *P* = 0.002) (Table 2). Similarly, Cox proportional hazards regression confirmed a sustained increased risk of MACE among male patients over 2 years (HR: 1.18, 95% CI: 1.06–1.32; log-rank *P* = 0.003) (Figure 2). The proportionality test yielded a non-significant *P*-value (*P* > 0.05) for MACE at both 1- and 2-year follow-up, supporting the appropriateness of the Cox proportional hazards model. In a sensitivity analysis redefining MACE as a composite of MI and stroke only, excluding all-cause mortality, male patients continued to demonstrate a significantly higher risk compared with female patients, consistent with the primary analysis (Supplementary Table S1).

**Table 2:** All-cause mortality and cardiovascular outcomes by sex.

Outcome	Male	Female	RR (95% CI)	*P* value	*E* value
1-year Outcomes					
MACE	558	437	1.22 (1.09–1.36)	0.003	1.74
All-cause mortality	492	431	1.12 (0.99–1.25)	0.054	1.49
MI	55	48	1.14 (0.78–1.68)	0.486	1.54
Stroke	58	41	1.40 (0.94–2.08)	0.092	2.15
Pericarditis	22	11	1.99 (0.97–4.10)	0.554	3.39
Acute heart failure	141	95	1.49 (1.15–1.92)	0.001	2.08
Atrial fibrillation	162	98	1.72 (1.35–2.20)	0.001	2.57
Ventricular arrhythmias	64	53	1.20 (0.84–1.73)	0.300	1.69
Torsade de pointes	32	36	0.88 (0.55–1.42)	0.620	1.28
High-grade AV block	94	46	2.08 (1.47–2.95)	0.001	3.53
Cardiomyopathy	85	63	1.36 (0.99–1.88)	0.056	1.86
2-year Outcomes					
MACE	697	593	1.15 (1.05–1.26)	0.002	1.43
All-cause mortality	665	569	1.18 (1.07–1.30)	0.004	1.56
MI	82	64	0.98 (0.69–1.38)	0.928	1.16
Stroke	67	47	1.41 (0.97–2.04)	0.060	2.17
Pericarditis	31	12	2.57 (1.32–4.99)	0.003	4.59
Acute heart failure	148	131	1.12 (0.89–1.41)	0.307	1.49
Atrial fibrillation	195	126	1.62 (1.30–2.01)	0.001	2.62
Ventricular arrhythmias	75	49	1.53 (1.07–2.18)	0.017	2.43
Torsade de pointes	35	43	0.81 (0.52–1.26)	0.354	1.23
High-grade AV block	101	56	1.84 (1.33–2.54)	0.001	3.02
Cardiomyopathy	100	82	1.23 (0.92–1.64)	0.150	1.78

MACE, major adverse cardiovascular events; MI, myocardial infarction; AV, atrioventricular.

### Secondary outcomes

At 1-year follow-up, all-cause mortality was numerically higher among male patients compared with females but did not reach statistical significance (492 vs. 431 events; RR: 1.12, 95% CI: 0.99–1.25; *P* = 0.054). The risk of stroke was also numerically higher among males but did not reach statistical significance (58 vs. 41 events; RR: 1.40, 95% CI: 0.94–2.08; *P* = 0.092). Male patients demonstrated significantly higher risks of acute heart failure (141 vs. 95 events; RR: 1.49, 95% CI: 1.15–1.92; *P* = 0.001), atrial fibrillation (162 vs. 98 events; RR: 1.72, 95% CI: 1.35–2.20; *P* = 0.001), and high-grade AV block (94 vs. 46 events; RR: 2.08, 95% CI: 1.47–2.95; *P* = 0.001). No significant sex-based differences were observed for MI (55 vs. 48 events), pericarditis (22 vs. 11 events), ventricular arrhythmias, torsade de pointes, or cardiomyopathy at 1 year.

At 2-year follow-up, all-cause mortality was significantly higher among male patients compared with females (665 vs. 569 events; RR: 1.18, 95% CI: 1.07–1.30; *P* = 0.004). The difference in stroke risk remained non-significant (67 vs. 47 events; RR 1.41, 95% CI: 0.97–2.04; *P* = 0.060). Male patients had significantly higher risks of atrial fibrillation (195 vs. 126 events; RR: 1.62, 95% CI: 1.30–2.01; *P* = 0.001), high-grade AV block (101 vs. 56 events; RR: 1.84, 95% CI: 1.33–2.54; *P* = 0.001), ventricular arrhythmias (75 vs. 49 events; RR: 1.53, 95% CI: 1.07–2.18; *P* = 0.017), and pericarditis (31 vs. 12 events; RR: 2.57, 95% CI: 1.32–4.99; *P* = 0.003). No statistically significant sex-based differences were observed for MI (82 vs. 64 events), acute heart failure, torsade de pointes, or cardiomyopathy at 2 years (Table 2).

## Discussion

In this large real-world, propensity-matched analysis, male patients undergoing CAR-T cell therapy had a significantly higher risk of MACE compared with female patients. At 2-year follow-up, male patients also had a higher risk of all-cause mortality. Additionally, males had higher rates of arrhythmic and conduction abnormalities, most notably atrial fibrillation and high-grade AV block, at both one- and two-year follow-up. Acute heart failure was more frequent among males at 1 year, although this association attenuated at 2 years. To our knowledge, this is among the first studies to identify a sex-based disparity in overall MACE following CAR-T therapy. Prior investigations have yielded mixed findings. An analysis using the National Readmissions Database did not identify statistically significant sex-based differences in cardiac complications; however, that study did not specifically evaluate cardiotoxicity, stratify individual MACE components, or assess longitudinal outcomes (13). Similarly, Lefebvre et al. did not observe a statistically significant association between sex and cardiotoxicity, although a higher incidence of cardiotoxic events was reported among patients who developed grade 3–4 CRS. These differences across studies may reflect variability in study design, outcome definitions, sample size, and duration of follow-up (14).

Consistent with eligibility criteria in pivotal CAR-T clinical trials, biological sex remains a well-established determinant of cardiovascular risk in the general population. Large population-based data from the EPIC-Norfolk study demonstrate that male sex is independently associated with a higher lifetime risk of heart failure, CAD, and atrial fibrillation, with earlier onset of cardiovascular events compared with females, even after adjustment for traditional risk factors (15). Importantly, these associations persisted after propensity score matching across a comprehensive set of baseline demographic, clinical, and cardiovascular characteristics, suggesting that the observed sex-based differences are not fully explained by baseline risk imbalances alone. Together, these findings suggest that an underlying differential susceptibility may extend to CAR-T–associated cardiotoxicity, potentially manifesting as a higher burden of arrhythmic and conduction abnormalities in males. The pathophysiology of CAR-T associated cardiotoxicity is closely linked to CRS. Prior mechanistic studies integrating preclinical and clinical data have demonstrated that CRS is present in nearly all cases of QTc prolongation and torsades de pointes, and in approximately 79% of patients with atrial tachyarrhythmias, including atrial fibrillation. In contrast, only about half of cardiomyopathy cases occur concurrently with CRS, suggesting that additional mechanisms such as direct myocardial injury and cytokine-mediated myocardial depression may also contribute. CAR-T cell activation following antigen recognition triggers a robust proinflammatory cytokine cascade, including IL-2, IL-6, IL-8, IL-10, TNF-α, and IFN-*γ*, with downstream activation of monocytes and macrophages (16–18). Elevated circulating IL-6 levels have been associated with QTc prolongation and long QT syndrome, increasing susceptibility to malignant ventricular arrhythmias such as torsades de pointes, and have also been linked to a higher risk of spontaneous ventricular tachyarrhythmias in patients with coronary artery disease. Mechanistically, cytokine-mediated alterations in cardiac ion channel activity, including increased I_Na_ density, may delay repolarization and promote electrical instability (19). Notably, ventricular arrhythmias emerged as a significant finding at 2 years among male patients, but not at 1 year, suggesting a delayed pro-arrhythmic effect that may reflect progressive cytokine-mediated myocardial remodeling. Sex-based differences in immune responses and inflammatory signaling may therefore partially explain the higher burden of arrhythmias and conduction abnormalities observed in males following CAR-T therapy.

Given that cardiovascular mortality following CAR-T therapy has been reported to range between 1.4% and 4.3%, our findings underscore the importance of incorporating sex into cardiovascular risk stratification prior to CAR-T initiation (20, 21). Male patients appear to be at higher risk for arrhythmic and conduction abnormalities and may benefit from enhanced cardiac surveillance during and after therapy. These findings suggest that biological sex may influence cardiovascular risk following CAR-T therapy; however, alternative explanations including differences in healthcare utilization, cardiovascular surveillance intensity, referral patterns, and treatment practices between male and female patients cannot be excluded. Accordingly, these results should be interpreted as hypothesis-generating rather than evidence of causation given the observational nature of the study design, and warrant confirmation in prospective studies. Nonetheless, these findings would support a sex-informed cardio-oncology approach, including baseline cardiovascular assessment, closer rhythm monitoring, and early multidisciplinary involvement to mitigate cardiac complications without compromising oncologic efficacy. E-values were calculated to quantify robustness to unmeasured confounding. The E-values for MACE ranged from 1.43 to 1.74 across follow-up timepoints, indicating that while some reassurance exists regarding the stability of our findings, relatively moderate unmeasured confounding could potentially explain the observed associations, and results should therefore be interpreted with appropriate caution.

A key strength of this study is the large, well-balanced cohort of CAR-T–treated patients, enabling robust sex-based comparisons in a real-world setting. The use of propensity score–matched analyses, together with evaluation of outcomes across both one- and two-year follow-up intervals, allowed assessment of the consistency and temporal persistence of cardiovascular risk after CAR-T therapy. In addition, the granular examination of individual components of major adverse cardiac events, including arrhythmic, conduction, and structural outcomes, provides clinically meaningful insight into the nature and durability of sex-based differences in CAR-T–associated cardiotoxicity. Despite these strengths, several limitations warrant consideration. The retrospective, observational nature of the study precludes causal inference, and all findings represent associations that should not be interpreted as causal effects. Outcome ascertainment based on ICD-10 codes introduces inherent risks of misclassification, underreporting, and inter-institutional coding variability, which may differentially affect event detection between sexes. Although anthracycline exposure, prior radiotherapy, CRS severity, ICANS, and baseline cancer type were incorporated into the propensity score model, several clinically important variables, including CAR-T product type, conditioning regimens, tumor burden, cancer stage, cardiotoxicity grading, and timing of cardiovascular events relative to CRS onset, were not consistently available within the TriNetX platform, limiting mechanistic interpretation. Disease relapse and subsequent cardiotoxic salvage therapies represent time-varying confounders that could not be accounted for, and residual confounding from unmeasured CAR-T–specific and oncologic variables cannot be fully excluded. While most covariates achieved adequate balance after matching, a small number had residual SMDs between 0.10 and 0.20. The inclusion of all-cause mortality in the MACE composite may incorporate non-cardiovascular deaths; however, a sensitivity analysis redefining MACE as MI and stroke only yielded consistent results, supporting the robustness of our primary findings. Secondary outcomes were exploratory and no correction for multiple comparisons was performed; these findings should therefore be interpreted with appropriate caution. Finally, all-cause mortality represents a competing risk for secondary cardiovascular outcomes not formally modeled in the Cox analyses; future studies incorporating competing risk frameworks such as the Fine-Gray model would further strengthen these findings.

## Conclusion

In this large real-world, propensity-matched analysis, male patients undergoing CAR-T cell therapy had a higher risk of MACE compared with female patients, along with higher rates of arrhythmic and conduction abnormalities. These associations were consistent across both short- and intermediate-term follow-up. While these findings are hypothesis-generating and should not be interpreted as causal, they suggest the potential importance of incorporating biological sex into cardiovascular risk assessment and surveillance strategies in patients receiving CAR-T therapy, and underscore the need for prospective studies to confirm and mechanistically explore these observations.

## Data Availability

The raw data supporting the conclusions of this article will be made available by the authors, without undue reservation.

## References

[1] Zugasti I Espinosa-Aroca L Fidyt K Mulens-Arias V Diaz-Beya M Juan M. CAR-T cell therapy for cancer: current challenges and future directions. Signal Transduct Target Ther. (2025) 10:210. 10.1038/s41392-025-02269-w 40610404 PMC12229403

[2] Maude SL Frey N Shaw PA Aplenc R Barrett DM Bunin NJ. Chimeric antigen receptor T cells for sustained remissions in leukemia. N Engl J Med. (2014) 371:1507–17. 10.1056/NEJMoa1407222 25317870 PMC4267531

[3] Ghosh AK Chen DH Guha A Mackenzie S Walker JM Roddie C. CAR T cell therapy-related cardiovascular outcomes and management: systemic disease or direct cardiotoxicity? JACC CardioOncol. (2020) 2:97–109. 10.1016/j.jaccao.2020.02.011 34396213 PMC8352125

[4] Gutierrez C Neilan TG Grover NS. How I approach optimization of patients at risk of cardiac and pulmonary complications after CAR T-cell therapy. Blood. (2023) 141:2452–9. 10.1182/blood.2022017579 36827628 PMC10329189

[5] Alvi RM Frigault MJ Fradley MG Jain MD Mahmood SS Awadalla M. Cardiovascular events among adults treated with chimeric antigen receptor T-cells (CAR-T). J Am Coll Cardiol. (2019) 74:3099–108. 10.1016/j.jacc.2019.10.038 31856966 PMC6938409

[6] Ganatra S Carver JR Hayek SS Ky B Leja MJ Lenihan DJ. Chimeric antigen receptor T-cell therapy for cancer and heart: JACC council perspectives. J Am Coll Cardiol. (2019) 74:3153–63. 10.1016/j.jacc.2019.10.049 31856973 PMC8211027

[7] Lyon AR Dent S Stanway S Earl H Brezden-Masley C Cohen-Solal A. Baseline cardiovascular risk assessment in cancer patients scheduled to receive cardiotoxic cancer therapies: a position statement and new risk assessment tools from the cardio-oncology study group of the heart failure association of the European society of cardiology in collaboration with the international cardio-oncology society. Eur J Heart Fail. (2020) 22:1945–60. 10.1002/ejhf.1920 32463967 PMC8019326

[8] Di Florio DN Sin J Coronado MJ Atwal PS Fairweather D. Sex differences in inflammation, redox biology, mitochondria and autoimmunity. Redox Biol. (2020) 31:101482. 10.1016/j.redox.2020.101482 32197947 PMC7212489

[9] Wilcox NS Rotz SJ Mullen M Song EJ Ky Hamilton B Moslehi J. Sex-specific cardiovascular risks of cancer and its therapies. Circ Res. (2022) 130:632–51. 10.1161/CIRCRESAHA.121.319901 35175846 PMC8915444

[10] Topaloglu U Palchuk MB. Using a federated network of real-world data to optimize clinical trials operations. JCO Clin Cancer Inform. (2018) 2:1–10. 10.1200/CCI.17.00067 30652541 PMC6816049

[11] Benchimol EI Smeeth L Guttmann A Harron K Moher D Petersen I. The REporting of studies conducted using observational routinely-collected health data (RECORD) statement. PLoS Med. (2015) 12:e1001885. 10.1371/journal.pmed.1001885 26440803 PMC4595218

[12] VanderWeele TJ Ding P. Sensitivity analysis in observational research: introducing the E-value. Ann Intern Med. (2017) 167:268–74. 10.7326/M16-2607 28693043

[13] Tan JY Yeo YH Kin HWK Ang QX Chisti MM Ezekwudo D. Sex differences in outcomes of chimeric antigen receptor (CAR) T-cell therapy. Cancer Med. (2025) 14:e70831. 10.1002/cam4.70831 40129265 PMC11933716

[14] Lefebvre B Kang Y Smith AM Frey NV Carver JR Scherrer-Crosbie M. Cardiovascular effects of CAR T cell therapy: a retrospective study. JACC CardioOncol. (2020) 2:193–203. 10.1016/j.jaccao.2020.04.012 32776016 PMC7413146

[15] Pana TA Mamas MA Wareham NJ Khaw KT Dawson DK Myint PK. Sex-specific lifetime risk of cardiovascular events: the European prospective investigation into cancer-norfolk prospective population cohort study. Eur J Prev Cardiol. (2024) 31:230–41. 10.1093/eurjpc/zwad283 38031203 PMC10809170

[16] Totzeck M Michel L Lin Y Herrmann J Rassaf T. Cardiotoxicity from chimeric antigen receptor-T cell therapy for advanced malignancies. Eur Heart J. (2022) 43:1928–40. 10.1093/eurheartj/ehac106 35257157 PMC9123242

[17] Du H Wang J Wang Z. Cardiovascular adverse effects of immunotherapy in cancer: insights and implications. Front Oncol. (2025) 15:1601808. 10.3389/fonc.2025.1601808 40606990 PMC12213269

[18] Ganatra S Redd R Hayek SS Parikh R Azam T Yanik GA. Chimeric antigen receptor T-cell therapy-associated cardiomyopathy in patients with refractory or relapsed non-hodgkin lymphoma. Circulation. (2020) 142:1687–90. 10.1161/CIRCULATIONAHA.120.048100 33104402

[19] Alí A Boutjdir M Aromolaran AS. Cardiolipotoxicity, inflammation, and arrhythmias: role for interleukin-6 molecular mechanisms. Front Physiol. (2018) 9:1866. 10.3389/fphys.2018.01866 30666212 PMC6330352

[20] Ganatra S Dani SS Yang EH Zaha VG Nohria A. Cardiotoxicity of T-cell antineoplastic therapies: JACC: cardioOncology primer. JACC CardioOncol. (2022) 4:616–23. 10.1016/j.jaccao.2022.07.014 36636447 PMC9830211

[21] Chen L-R Li Y-J Zhang Z Wang P Zhou T Qian K. Cardiovascular effects associated with chimeric antigen receptor T cell therapy in cancer patients: a meta-analysis. Front Oncol. (2022) 12:924208. 10.3389/fonc.2022.924208 36439485 PMC9682079

