# Supplementary material for: Sex-based disparities in cardiovascular outcomes: real-world evidence following chimeric antigen receptor T-cell therapy
Source: Front Cardiovasc Med. 2026 Jul 8;13:1838296. doi: 10.3389/fcvm.2026.1838296 (PMC13388846; doi:10.3389/fcvm.2026.1838296)
Supplement: Supplementary file 1 [file Table1.docx]

**Supplementary Appendix**

**Title: Sex-based Disparities in Cardiovascular Outcomes: Real-world Evidence Following Chimeric Antigen Receptor T-Cell Therapy**

**Table S1**: Sensitivity Analysis of MACE Excluding All-Cause Mortality

| **Outcome** | **Male** | **Female** | **RR (95% CI)** | **P value** | **E value** |
| --- | --- | --- | --- | --- | --- |
| **1-year Outcome** | | | | | |
| MACE | 132 | 90 | 1.11 (1.03–1.34) | 0.04 | 1.46 |
| **2-year Outcome** | | | | | |
| MACE | 146 | 107 | 1.09 (1.01–1.23) | 0.048 | 1.40 |
